# Polymer Hernia Repair Materials: Adapting to Patient Needs and Surgical Techniques

**DOI:** 10.3390/ma14112790

**Published:** 2021-05-24

**Authors:** Marta Rodríguez, Verónica Gómez-Gil, Bárbara Pérez-Köhler, Gemma Pascual, Juan Manuel Bellón

**Affiliations:** 1 Departamento de Cirugía, Ciencias Médicas y Sociales, Facultad de Medicina y Ciencias de la Salud, Universidad de Alcalá, Alcalá de Henares, 28805 Madrid, Spain; marta.rodriguezma@uah.es (M.R.); veronica.gomezg@uah.es (V.G.-G.); 2 Biomedical Networking Research Centre of Bioengineering, Biomaterials and Nanomedicine (CIBER-BBN), 28029 Madrid, España; barbara.perez@uah.es (B.P.-K.); gemma.pascual@uah.es (G.P.); 3 Ramón y Cajal Health Research Institute (IRYCIS), Colmenar Viejo, 28034 Madrid, Spain; 4 Departamento de Medicina y Especialidades Médicas, Facultad de Medicina y Ciencias de la Salud, Universidad de Alcalá, Alcalá de Henares, 28805 Madrid, Spain

**Keywords:** Polypropylene, polytetrafluoroethylene, meshes

## Abstract

Biomaterials and their applications are perhaps among the most dynamic areas of research within the field of biomedicine. Any advance in this topic translates to an improved quality of life for recipient patients. One application of a biomaterial is the repair of an abdominal wall defect whether congenital or acquired. In the great majority of cases requiring surgery, the defect takes the form of a hernia. Over the past few years, biomaterials designed with this purpose in mind have been gradually evolving in parallel with new developments in the different surgical techniques. In consequence, the classic polymer prosthetic materials have been the starting point for structural modifications or new prototypes that have always strived to accommodate patients’ needs. This evolving process has pursued both improvements in the wound repair process depending on the implant interface in the host and in the material’s mechanical properties at the repair site. This last factor is important considering that this site—the abdominal wall—is a dynamic structure subjected to considerable mechanical demands. This review aims to provide a narrative overview of the different biomaterials that have been gradually introduced over the years, along with their modifications as new surgical techniques have unfolded.

## 1. Introduction

The spectacular rise in the use of biomaterials in clinical practice has meant that prophylactic materials today play a major role in the development of surgical techniques in all medical specialties [1]. The field of biomaterials and their applications is perhaps the most dynamic of all advanced technological developments. As one of their multiple applications, these materials are invariably used to strengthen or replace defective abdominal wall tissues such as when repairing a hernia.

The term hernia refers to the abnormal protrusion of an organ or part of an organ outside the body cavity in which it is normally contained. Hernias most often arise in the abdomen, causing pain or discomfort to the patient and limiting daily activity. To mechanically close the hernial cavity and reinforce the abdominal wall, the standard surgical technique is synthetic mesh placement. Currently, more than 20 million hernias are operated on each year across the world [2]. In the United States alone, some 700,000 inguinal hernia operations are performed every year. The frequency of incisional hernia, i.e., a hernia produced as a consequence of a prior surgical incision weakening the abdominal wall, is also remarkably high [3,4]. In some cases, the objective of surgery using a prosthetic material is to repair defects generated when a tumour or metastasis is excised, as a malignancy in the peritoneal cavity may invade the abdominal wall [5].

Based on clinical evidence, the use of a prosthetic material is currently recommended for the repair of a hernia, whether this is a primary defect (primary hernia) or the consequence of a prior laparotomy (incisional hernia) [6,7,8,9]. Mesh hernia repair thus replaced the traditional suture closure techniques. The free-tension repair concept of Lichtenstein et al. [10], advocating the use of a mesh or patch to repair a hernia revolutionised all surgical procedures designed to repair an abdominal wall defect. The same has occurred with the repair of incisional hernias, in which the use of a biomaterial is today almost mandatory and this has served to reduce recurrence rates [11]. As early as in 1960, Usher [12] heralded what was later to be promulgated and popularized by Lichtenstein’s group: “if mesh is used to bridge the defect instead of reinforcement for tissues approximated under stress, this factor of tension is eliminated, and recurrence becomes less likely…”.

Abdominal wall repair is a challenging and complex procedure that includes the reconstruction of the original tissue structure and restoration of its previous function. The abdominal wall comprises distinct layers whose integrity has to be maintained. The recovery of the elasticity and natural strength of the abdomen must be guaranteed as well after abdominal wall reconstruction. Research and development of biomaterials to be used in the repair of abdominal wall defects is thus an ever-expanding field. Their use in the past 20 years has conditioned these prosthetic materials, which have gradually been modified in an effort to develop a biomaterial that shows optimal behaviour at every tissue interface.

Developments in second and third generation materials that take into account the recipient organism and its biology to improve their host tissue integration is effectively an attractive area of research. Similarly, the development of materials for this purpose has had to constantly adapt to new surgical techniques such as laparoscopic surgery. Therefore, there is a vast variety of prosthetic materials with different properties and indications available for abdominal wall repair.

The objective of this report is to provide a narrative overview of the different biomaterials that have been gradually introduced over the years, along with their modifications and their adaptation to surgical advances made in hernia repair.

## 2. Classic Polymer Biomaterials and Hernia Repair

The three biomaterials that have been milestones in the field of hernia repair that are still used today are: polyester, or Dacron mesh (Mersilene^®^), polypropylene (PP) mesh (Marlex^®^) and expanded polytetrafluoroethylene (ePTFE) mesh (Soft Tissue Patch^®^).

As early as 1956, Dacron^®^ fabric started to be used for inguinal and ventral hernia repair. The first study, conducted by Wolstenholme [13] gave rise to promising results as patients’ hernial defects were treated without great complications.

In a review conducted in 1975, Stoppa et al. [14] highlighted the benefits of Dacron^®^ mesh when used to repair recurrent giant groin hernias. These authors argued that, when adequately placed in the preperitoneal space, this mesh acts as a non-resorbable artificial endoabdominal fascia, instantly conferring lasting strength to the abdominal wall. Wantz in 1991 [15] confirmed the good results obtained with this material. The Dacron mesh was the first non-metal prosthesis to be widely incorporated into clinical practice although its use started to decline as PP mesh gained popularity.

The first PP mesh marketed under the name of Marlex^®^ was introduced by Usher in 1959 [16]. This mesh featured several benefits over the metal meshes used at the time, as it was much more flexible and could be easily inserted into a defect of any size without fragmenting like the metal meshes. It also seemed more resistant to infection. Two years later, Usher described the use of a Marlex^®^ prosthesis to bridge lesions in the abdominal wall, with good outcomes in terms of low recurrence rates (10.2% for incisional hernia, 5.9% for inguinal) [17].

Given its advantages, the popularity of Marlex^®^ rapidly spread. In 1961, Usher [18] described an improved version of Marlex^®^ comprising a mesh woven from polypropylene monofilament suture thread. Later studies confirmed the benefits of hernia repair using Marlex^®^ [19,20,21,22,23]. In 1989, Lichtenstein [24] reported his good results with Marlex^®^ used in 1000 patients with inguinal hernia.

Already in the 1990s, several techniques were developed to repair large incisional hernias in the abdominal wall, while sparing the peritoneum between the organs and mesh. Outcomes were satisfactory in terms of recurrence rates, and while infection was observed in a small proportion, no prosthesis had to be removed [25,26,27].

The third classic prosthetic material used to repair a hernial defect was polytetrafluoroethylene (PTFE). The first report of the use of PTFE (Teflon) for the repair of an abdominal wall defect was that by Harrison in 1957 [28] in which results were promising. However, when this same material was woven to generate a prosthesis, outcomes were disappointing and it was discontinued [29].

In 1967, Oshige [30] described a process whereby PTFE could be expanded to modify its microstructure and achieve greater mechanical strength. This technique was refined by the company Gore and Associates [31] and clinically applied to vascular prostheses. Following this use, PTFE was radically expanded to generate a sheet material that could be used to repair hernias and other soft tissue defects. It was named the Soft Tissue Patch^®^ and introduced for the first time in clinical practice in 1983.

Just as PTFE, expanded PTFE (ePTFE) is inert in tissues and induces a scarce foreign body reaction in the host. The Soft Tissue Patch^®^ is manufactured as sheets of different calibres and a thickness of 1 or 2 mm. It is comprised of nodes of PTFE forming columns connected by fine PTFE fibrils, which are multidirectionally angled on the surface. This confers the mesh balanced resistance properties in all directions. Mean internodal fibril length, or pore size, is 20 to 25 μm, and this unique porous structure offers a flexible biomaterial that is soft and easily handled, does not fray and allows for cell infiltration.

Studies have shown that ePTFE has an adequate tensile strength for its safe clinical use. Through industrial testing methods, it has been proven stronger than the meshes Marlex or Dacron and similar to these materials in terms of suture retention resistance.

In 1979, initial experimental investigations [21] revealed the good biological tolerance of this material. Sher et al. in 1980 [32] confirmed for the first time its good behaviour at the peritoneal interface in relation to polypropylene. These findings were highlighted by Lamb et al. [33], who confirmed that the peritoneal reaction to the implants was minimal.

After 1985, the first clinical trials on the use of ePTFE offered good results in both the short and long terms. There were barely any recurrences, infections or surgical complications, and it was thus concluded that this prosthetic material was perfectly tolerated by the human body [34,35,36,37,38]. This was a great advance, as it was associated with a lower incidence of adhesions, which had so far been one of the major shortcomings of the materials available. Further benefits were good integration of the prosthetic mesh in the host tissue and the development in experimental animals of a continuous layer of mesothelial cells on the side of the mesh in contact with the peritoneum by the fourth week post-implant [39,40].

In 1992, de Bord et al. [41] published their findings in a study in which 62 patients with large incisional hernia underwent repair with Soft Tissue Patch^®^. The recurrence rate recorded in this patient series was 12.9%.

In 1993, Berliner [42] described his experience with the treatment of 350 inguinal hernias with an ePTFE soft tissue patch for tension-free repair under local anaesthesia in an ambulatory setting. During a mean follow up of 41.8 months, there were four recurrences (1.1%). Graft infection was a mere 0.29%, although a persistent fistula required patch removal.

In 1997, Bellón et al. [43] related their experience with the repair of large groin hernias using an ePTFE patch in 38 patients. After a follow up ranging from 18 to 72 months, three recurrences (7.8%) and one episode of post-implant intestinal obstruction were recorded.

### 2.1. Structural Modifications to the Classic Polymer Biomaterials

Since the 1990s, these classic biomaterials have undergone modifications targeted at improving the mesh/host tissue interface for both better host tissue incorporation and mechanical strength.

The first structural modifications were made to the ePTFE prosthetics and the starting point was the Soft Tissue Patch^®^.

These modifications to the soft tissue patch gave rise to Mycro Mesh^®^. This macroporous mesh consists of a standard microporous mesh with evenly spaced large pores for more rapid tissue incorporation in the prosthesis [44]. The second variation is Dual Mesh^®^, which is made up of two surfaces, a non-porous side designed to avoid adhesion formation, and a standard microporous surface to allow for host tissue incorporation [45]. This latter surface was subjected to further modification to create a rougher surface for better host tissue ingrowth (Dual Mesh Corduroy^®^). Another development was the pretreatment of the prosthetic mesh surface with an antibacterial agent (silver and chlorhexidine) giving rise to the Dual Mesh Plus^®^. The result was an antibacterial prosthetic material designed to avoid the adherence of bacteria. This was the first antibacterial mesh to be commercialized. Studies in vitro have confirmed the benefits provided by this pretreatment [46].

The most recent modification to an ePTFE prosthesis has been the creation of a reticular non-expanded PTFE mesh (Infinit Mesh^®^). The idea pursued was adequate host tissue incorporation to improve the strength of the repair zone putting this mesh in competition with the lightweight and heavyweight PP prostheses. Experimental findings have indicated no difference in tissue incorporation in relation to conventional PP mesh [47,48].

**Figure 1:**
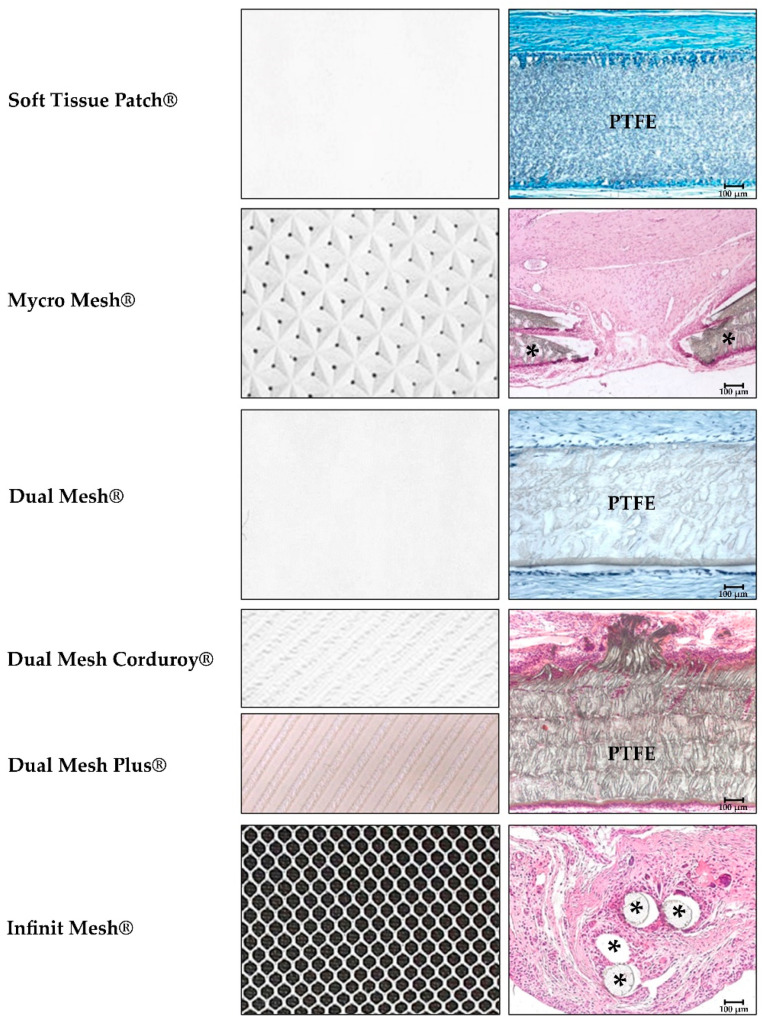
Macroscopic images of the different modifications in polytetrafluoroethylene (PTFE) meshes (**left**) and host tissue incorporation once implanted (**right**). Both surfaces, subcutaneous and peritoneal sides of the PTFE implants (Soft tissue Patch^®^, Dual mesh^®^, Dual Mesh Corduroy^®^-Dual Mesh Plus^®^, 30, 14, and 90 days post-implant, respectively, 100×) were encapsulated by host connective tissue. Scar tissue surrounds the PTFE implants, and some cells could be seen into the prosthetic interstices, at the inner third of the PTFE. Furthermore, in Mycro Mesh^®^, host tissue penetrates through the material micropores (60 days post-implant, 100×). Infinit Mesh^®^ behaviour was similar to reticular meshes integration, like polypropylene, with connective tissue surrounding the mesh filaments (14 days post-implant, 100×). Scale bar: 100 µm.

Before this PTFE design, a similar design had been described in the literature but of ePTFE, with which good mechanical results had been obtained following its implant in the host [49] (Figure 1).

Polypropylene prostheses were also subjected to structural changes, and the starting point was always the classic prosthesis Marlex^®^. In the newer designs, factors were considered such as pore size, prosthetic filament diameter and the spatial distribution of filaments [50]. The pores of the classic PP designs were enlarged in size to attain diameters exceeding 1 mm and giving rise to the lightweight meshes of lower density or g/m^2^ of material [51].

This led to classification schemes whereby the classic PP meshes with a density of 80 g/m^2^ were considered heavyweight while materials of lower density to this threshold were classified as lightweight [52,53]. This was later to be followed by the introduction of materials of intermediate density ranging between 50 and 80 g/m^2^, determining that meshes are presently described as lightweight when their density is lower than 50 g/m^2^ [54].

Sometimes prosthetic weight is independent of pore size. Hence, implant materials with small pores and a simple spatial structure involving crossovers or knots comprised of a very fine filament can still be of fairly low density [55]. This aspect is important, as in agreement with the German school of thought [56], pore size has been the main factor used to describe a prosthetic material as of high or low density determining that implants described as high-density always have pores smaller than 1 mm, while low-density ones have a pore size larger than 1 mm.

Another modification employing PP as the structural basis has taken the form of hybrid or partially absorbable prosthetic devices. In these, polypropylene filaments are intermeshed with absorbable filaments. The hybrid materials are low density with large pores [57]. The absorbable component was initially a polyglactin polymer (Vypro^®^) but was later replaced by polyglecaprone (Ultrapro^®^).

Another innovation has been the pretreatment of PP meshes. For this purpose, a titanium coating has been the most widely used [58].

### 2.2. Modifications to Improve Host Tissue Incorporation

#### 2.2.1. Expanded Polytetrafluoroethylene

Because of their laminar structure, the host tissue incorporation achieved by ePTFE meshes at a tissue/tissue interface is deficient. Recipient tissue encapsulates these sheet prostheses with connective tissue. Further, as they are microporous, colonization is only cellular and there is scarce angiogenesis elicited. All this affects the mechanical strength of these implants which is particularly poor in zones of mesh anchorage to the host tissue [59].

With the aim to improve integration within host tissue and thus mechanical outcomes arose the first modification of introducing microperforations in the original Soft Tissue Patch^®^. Modifications were also made to its surface making it rough on one of its sides to generate Dual Mesh^®^. In both cases, no improvement was noted in terms of mechanical strength compared with the initial patch [60,61].

The genesis of a prosthesis in the form of a mesh (Infinit^®^) [62] elaborated from non-expanded polytetrafluoroethylene gave rise to both improved tissue incorporation and mechanical strength, although the elastic modulus of this material was excessively high [63]. Finally, the antibacterial ePTFE meshes have had scarce repercussions in clinical practice.

Contrary to what occurs at the tissue/tissue interface, ePTFE biomaterials such as the Soft Tissue Patch^®^ or DualMesh^®^ show excellent behaviour when placed directly in contact with the contents of the peritoneal cavity. Studies both in vitro and in vivo examining the formation of a neoperitoneum on the implanted prosthetic surface in contact with the intestinal loops have shown that the characteristics of this new layer depend upon the structure of the biomaterial employed for tissue repair [64].

**Figure 2:**
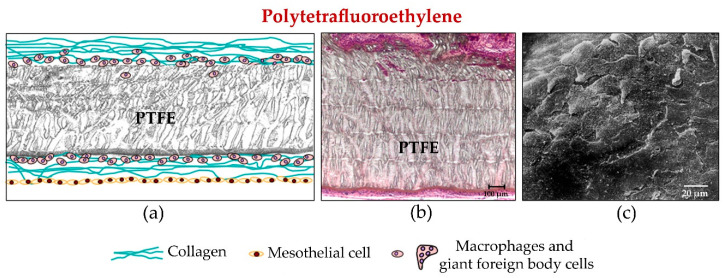
(**a**) Diagram and (**b**) light microscopy (90 days post-implant, 100×) images showing tissue incorporation (cross-section), in PTFE meshes, once implanted in the abdominal wall. Meshes are encapsulated by vascularized connective tissue arranged as fibrous bundles running parallel to the prosthetic surface. Cells are observed inside the biomaterial, although they fail to penetrate beyond the outer third of the laminar sheet. Scale bar: 100 µm. (**c**) Scanning electron microscopy view of mesothelial covering (14 days, 500×). Scale bar: 20 µm.

In experimental studies designed to monitor the prosthetic peritoneal surface following implant, a network of collagen fibres covered with typical mesothelial cells can be observed at an early stage. These fibres arrange themselves so that they run parallel to the prosthetic surface and are accompanied by a large number of cells, mostly fibroblasts and some foreign body reaction cells. In later stages, the neoperitoneum is remodelled and fibroblasts become the dominant cells at the expense of most of the foreign body reaction cells, which indicates good tolerance to the prosthesis. Finally, the collagen fibres organize themselves to run parallel to the implant surface, with the neoperitoneum on their outside making contact with the visceral peritoneum [65] (Figure 1 and Figure 2).

This perfectly configured neoperitoneum avoids one of the major complications that can arise following the implant of a biomaterial in contact with the visceral peritoneum, i.e., the formation of adhesions between the mesh and intestinal loops. Because of this behaviour, ePTFE meshes have been employed since the introduction of laparoscopic surgery for hernia repair [66,67,68]. In this type of surgery, the biomaterial is placed in direct contact with the contents of the abdominal cavity. This means that this interface needs to be as smooth as possible (to avoid inducing adhesions) by promoting adequate mesothelial deposition.

#### 2.2.2. Polypropylene

**Figure 3:**
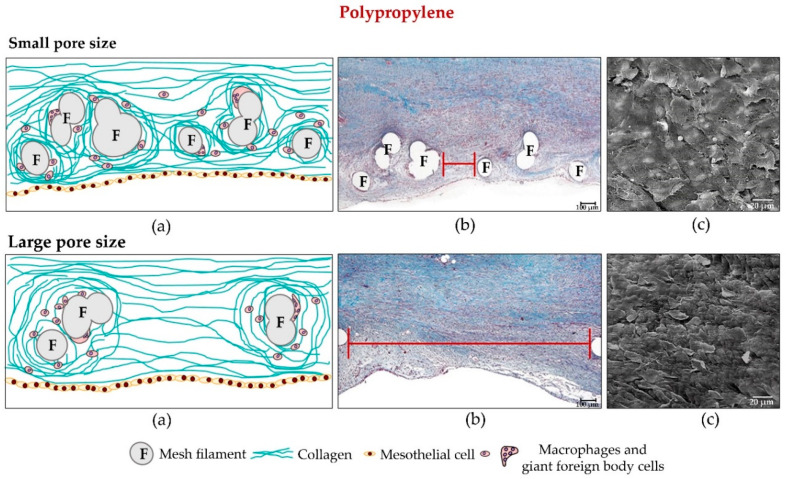
(**a**) Diagrams and (**b**) light microscopy images (100×) showing tissue incorporation (cross-section) in polypropylene meshes, once implanted in the abdominal wall (90 days post-implant). The prosthetic filaments are surrounded by scar tissue in which the collagen fibres concentrically lay around the mesh filaments. The spaces between filaments are also occupied by scar tissue. Scale bar: 100 µm. (**c**) Scanning electron microscopy view of mesothelial covering (14 days, 500×). Scale bar: 20 µm.

**Figure 4:**
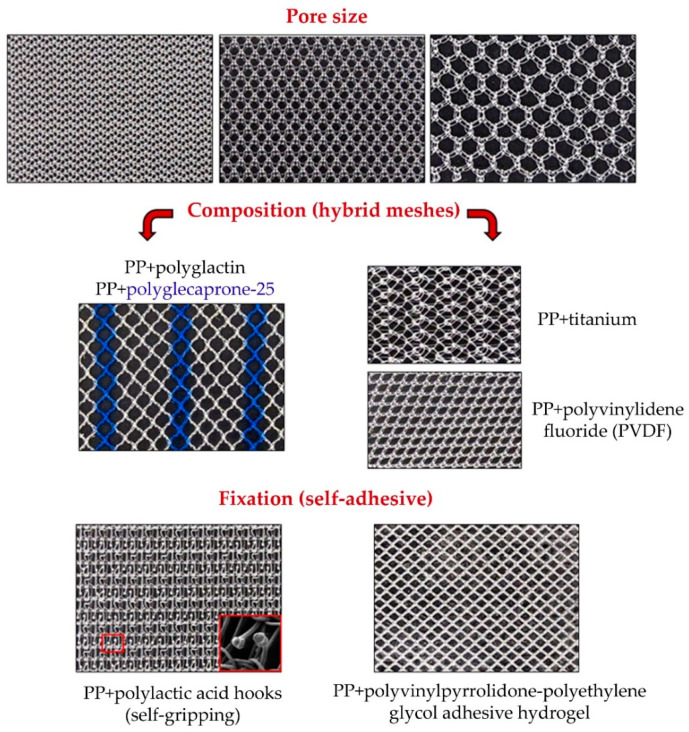
Macroscopic images of the different modifications to polypropylene meshes. Pore size: modifications in pore size have the objectives of minimizing minimize the quantity of foreign material in the host tissue, and improve the foreign body reaction and fibrosis without compromising mechanical resistance. Composition: hybrid meshes combine different components knitted or woven together to obtain a single mesh structure. Some of them incorporate an absorbable component (polyglecaprone-25 or polyglactin) to diminish the fibrosis reaction and amount of foreign material left in the body. Others include titanium or polymers like polyvinylidene fluoride. Fixation: self-adhesive meshes strive to achieve atraumatic mesh fixation (red box: polylactic acid hooks, scanning electron microscopy, 16×).

The rationale for the new low-density PP mesh designs was to minimize the foreign material implanted in the host in an effort to reduce the amount of fibrosis produced [69,70]. The idea was to avoid the abdominal rigidity, or lack of compliance, problems observed in some patients implanted with the conventional PP meshes, especially the high-density ones (i.e., those of small pore size). There is no doubt that reducing the final amount of foreign material left in the host should have considerable benefits, especially in younger patients (Figure 3 and Figure 4).

Studies conducted by our group [71] have shown that the tissue incorporation and mechanical strength offered by both the lightweight implants and the partially absorbable ones are similar to those of the conventional heavyweight reticular meshes. We should underscore that from the first moments of implant (2 weeks), collagen deposition can be detected on the large-pore implants [72,73]. This could explain why no differences exist in mechanical strength between low- and high-density materials when this factor is examined in the long term, i.e., 6 months after implant. In a recent study we observed that it is the recipient tissue that conditions implant behaviour in the long term, as similar mechanical strength values are obtained when comparing light- and heavyweight prosthetic meshes [74].

However, at the peritoneal interface, where these PP implants are in contact with the contents of the peritoneal cavity, the neoperitoneum generated is of a disorganized structure with a rough texture and zones of haemorrhage and necrosis which will further promote the appearance of adhesions [75]. We would thus argue that the reticular structure of this material leads to the inappropriate disposition of mesothelial cells on its surface.

Such behaviour patterns can be confirmed in in vitro experiments in which, after the seeding of mesothelial cells on different biomaterials, uniform rapid mesothelialization is only achievable with a laminar sheet material [76]. Seeding mesothelial cells on reticular PTFE has the same effect. Thus, it seems that the structure of a material, rather than its chemical composition, will condition its behaviour at the peritoneal level [49].

The birth of hybrid or partially absorbable prosthetics whose polymer base component is polypropylene has attempted to reduce even further the amount of foreign material left behind in the host after its implant. All these prosthetic materials are low density materials and their host tissue incorporation is similar to that of conventional PP.

With the objective of improving the biocompatibility of PP, this polymer is coated with titanium (Figure 4). The results obtained, however, both experimental and clinical, have been a matter of controversy. Thus, some authors have detected no benefits in preclinical studies of this PP treatment [77], while others argue that the foreign body reaction elicited in the host is diminished when titanium is incorporated into the PP [78,79]. In patients implanted with treated PP, some benefits seem to exist in terms of reduced postoperative pain and a more rapid recovery process [80,81].

### 2.3. Modifications Designed to Improve Adhesion to the Host: Self-Gripping Meshes

To improve mesh fixation to the host tissue, materials have been developed that have systems such as grips [82] or adhesives [83,84] to anchor the mesh. The objective of these designs has been to avoid the trauma of the use of sutures or tacks [85]. The idea behind these self-fixing meshes is to facilitate their placement at the repair site and shorten the time needed to do this.

The first of these meshes was Progrip^®^, a self-gripping mesh made of a low-weight knitted PP fabric (initially was made of polyester) that incorporates reabsorbable polylactic acid microhooks. These microhooks provide tissue-gripping properties of the mesh over the following 12 months [86].

The second mesh Adhesix^®^ is a self-adhesive, double-sided mesh, made of two components. A knitted monofilament PP mesh (rough side) covered by a reabsorbable layer of polyethylene glycol and polyvinylpyrrolidone (smooth side) [87]. These two components form a hydrogel that cross-links to the underlying tissue within 5 min. According to the manufacturer, the bioadhesive is reabsorbed within 7 days of implant. Mesh density after the reabsorption of both components is 40 g/m^2^.

Experimental and clinical outcomes of the use of these self-fixing meshes have been good overall both in terms of their host tissue incorporation and biomechanics [88,89,90,91,92] (Figure 4).

### 2.4. Reticular Polyvinylidenfluoride (PVDF) Materials

Among the reticular meshes, we find those fashioned out of polyvinylidenfluoride (PVDF) [93]. This polymer shows improved textile and biological properties. It is thermally stable and has been established as a suture material in cardiovascular and orthopaedic surgery applications [94]. Compared to other polymers such as polyester, it is more resistant to hydrolysis and degradation. Reports also exist of a diminished inflammatory response to this polymer [95]. The first mesh made of PVDF was promoted by the German research group of Schumpelick [96]. Notwithstanding, results obtained post-implant with this prosthesis, both preclinical and clinical, have been controversial, particularly when this material is used at a peritoneal interface [97,98,99,100,101,102,103] (Figure 4).

### 2.5. Condensed Polytetrafluoroethylene (cPTFE)

This is a non-woven, macroporous material that is manufactured through a PTFE condensing process. Its objectives have been to achieve good peritoneal behaviour including minimal adhesion formation and bacterial adherence.

Some preclinical studies have confirmed the improved performance of this mesh over that of ePTFE at the peritoneal interface [104,105]. Other studies, also experimental, while again describing the formation of fewer peritoneal adhesions, have detected risks associated with its intraperitoneal implant, especially regarding its peripheral zones [106]. In clinical practice, this mesh has been tested in a low number of patients with infection of the abdominal wall and results have been acceptable [107].

Table 1 summarizes the most representative modifications introduced in the polymeric materials employed in hernia repair.

## 3. Composite Materials

### 3.1. Classic Composite Materials

The different tissue behaviour of the classic biomaterials PP and ePTFE, especially when implanted at the tissue/tissue and peritoneal interface, has driven the search for a prosthetic material that encompasses the good qualities of both these materials. This led to the compound prostheses known as composites. In this combined prosthesis, the basic requirements of a prosthetic material proposed by Schein et al. [108] could be fulfilled: (a) elicit good host tissue ingrowth, (b) behave well at the peritoneal level, (c) and show good mechanical strength post-implant.

These prosthetic materials have two components. One of these is generally of reticular structure and designed to show good host tissue incorporation and the other, of smoother sheet texture, is designed to offer a good peritoneal interface.

Both components are usually joined together through acrylic adhesive, heat-sealing or even suture [109]. The reticular component was initially PP and subsequently it was polyester.

**Figure 5:**
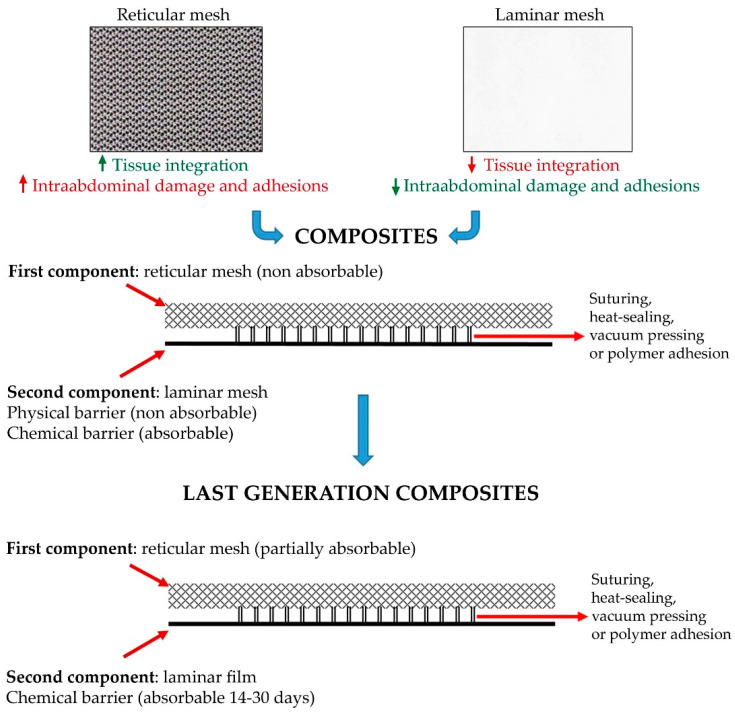
Composites consist of the combination of two different components linked together by suturing, heat-sealing, vacuum pressing or polymer adhesion.

The visceral contact component may be absorbable or non-absorbable. When non-absorbable, this component is known as a physical barrier [110,111,112] and when absorbable as a chemical barrier [113,114,115,116,117,118,119,120,121,122,123,124,125,126] (Figure 5).

The barriers used for visceral contact have always shared the structural characteristic of their smooth surface. Such a smooth surface facilitates the deposition and expansion of the mesothelial cells of the peritoneum. If the visceral contact surface is reticular, mesothelial cells are deposited incorrectly and this generates visceral adhesions [127].

**Figure 6:**
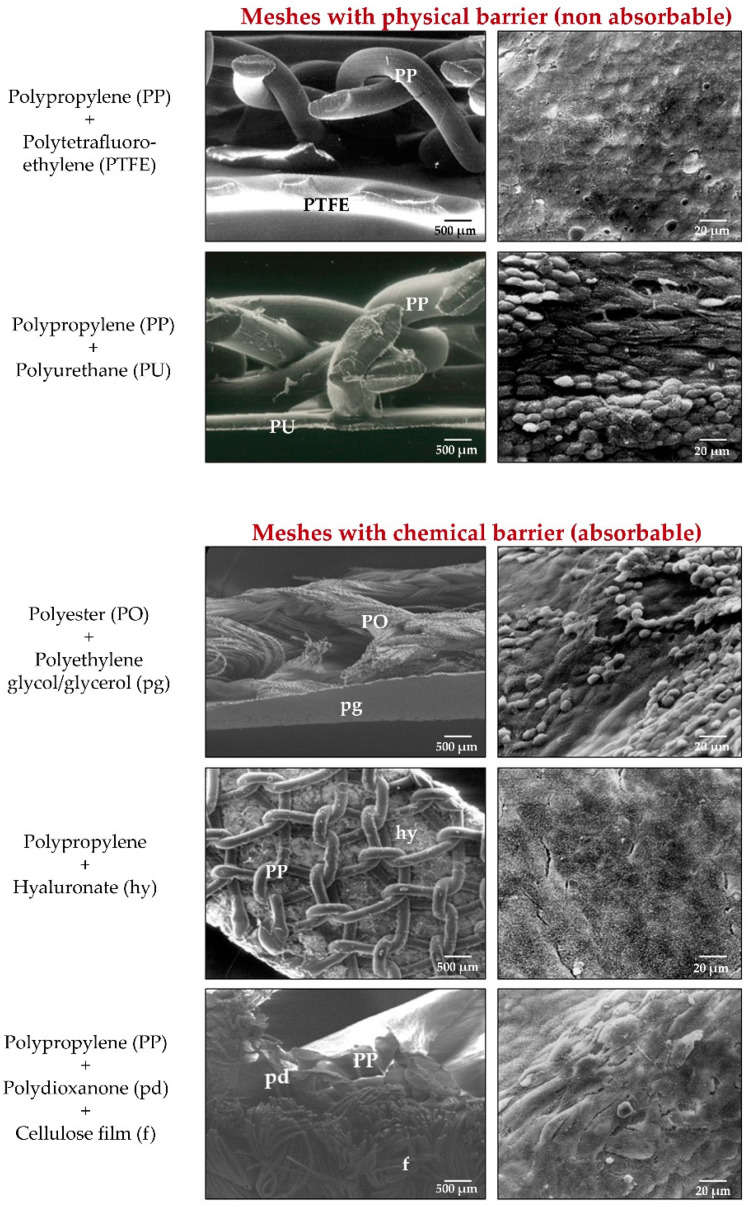
Scanning electron microscopy images of classic composites meshes (**left**) (lateral view, 50×. Scale bar: 500 µm) and mesothelial layer at the peritoneal side after implant (**right**, 500×. Scale bar: 20 µm). Composites include laminar components as adhesion barriers of physical (non absorbable) or chemical (absorbable) nature.

Physical and thus non biodegradable barriers were initially made of laminar PP or ePTFE. Other biomaterials employed were polyurethane [128,129] and silicone. As chemical barriers, collagen coated with polyethylenglycol/glycerol, and sodium hyaluronate have been employed (Figure 6).

The benefits of the absorbable components are that any type of adhesion arising after implant could hypothetically disappear with their degradation to give rise to a perfectly adequate peritoneal interface [130,131,132]. In general, whether biodegradable or not, these materials placed in contact with the visceral peritoneum should induce a minimal inflammatory reaction and allow for rapid and complete mesothelial cover [133].

Composites need to fulfill two objectives. The first is good integration within the host tissue and the second, for which they have been mainly designed, is to elicit adequate mesothelialization at the peritoneal level. This way, complications arising from the implant of a reticular material such as adhesions causing intestinal obstruction [134], implant migration to hollow organs [135], or very serious complications such as intestinal fistula [136,137], can be avoided.

Composite biomaterials are indicated for clinical use, mainly in open and laparoscopic repair surgery. Their tissue incorporation is improved over that achieved with the laminar ePTFE meshes. Clinical trials on prosthetic materials with a biodegradable chemical barrier have shown their good behaviour at the peritoneal interface [138,139,140,141,142].

While this peritoneal behaviour of composites is adequate, adhesions almost invariably form. On the upside, however, these adhesions are usually loose and easy to dissect or section. They are never integrated within the viscera (Table 2).

### 3.2. Structural Modifications to Classic Composite Materials

As composite materials have evolved in terms of their visceral contact component, in parallel the part designed for tissue integration has also advanced [143]. Thus, in the new prosthetic designs, the prosthetic component whose mission is to anchor the mesh in the host tissue has evolved from non-absorbable to absorbable. The objective pursued by these designs is to leave the least amount of foreign material possible in the recipient. In addition, the biomaterial initially acts as a scaffold so that host tissue will gradually invade the mesh and replace it as it gradually biodegrades for true tissue regeneration [144,145].

**Figure 7:**
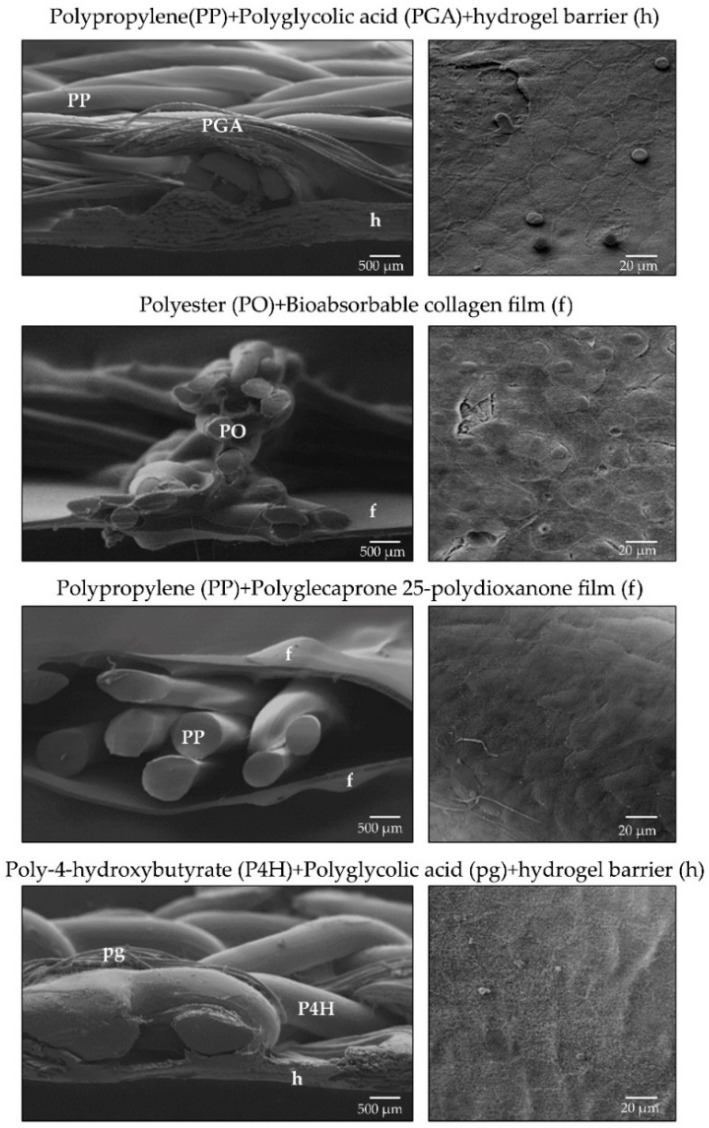
Scanning electron microscopy images of last generation composites (**left**) (lateral view, 50×. Scale bar: 500 µm) and mesothelial layer at the peritoneal side after implant (**right**, 500×. Scale bar: 20 µm). Nowadays, there is a tendency towards the use of reticular absorbable or partially absorbable components, and a short-term (14–30 days post-implant) absorbable laminar structure as adhesion barrier.

The materials used in these composites as the integrating component have been PP, 3D polyester, PP mesh coated with polyglecaprone 25 (partially absorbable), and poly-4-hydroxybutyrate (totally absorbable). On the visceral-facing side, the barriers, all chemical, have been polydioxanone, polyglycolic acid hydrogels and collagen with chitosan (Table 2, Figure 7).

In preclinical studies, the behaviour of these materials has emerged as appropriate and similar to that of the classic composites [146].

Recently, a new composite mesh has been introduced whose structure comprises low-density PP and a biological material composed of porcine intestinal submucosa. This material has been tested in clinical practice, though with a very short follow up, offering acceptable results [147].

## 4. Last-Generation Polymer Materials

**Figure 8:**
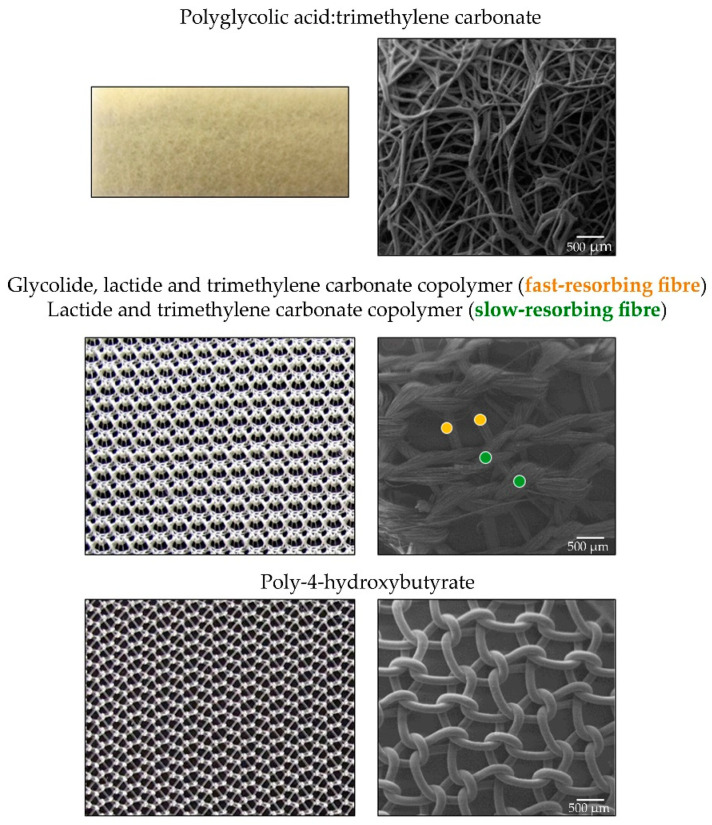
Research in new synthetic meshes are giving rise to fully absorbable products, like biocompatible synthetic polymers that are gradually absorbed by the host (macroscopic -left- and scanning electron microscopy images-right, 50×). Scale bar: 500 µm.

The last few years have seen the emergence of polymer materials that are fully biodegradable in the mid/long term with applications in hernia repair. These materials have the objective of reducing the foreign body reaction in the host and of promoting tissue regeneration (Figure 8).

One of the first to arise has been a compound of polyglycolic acid and trimethylene carbonate (Bio-A^®^) [148]. These polymers are widely known for their biocompatibility and while they have been used in the field of sutures in particular, experience to date with this prosthesis has been scarce.

Preclinical studies [149] have revealed the full biodegradation of this material in 3 to 6 months. In clinical practice [150], high recurrence rates have been detected when using Bio-A^®^ for the repair of inguinal hernias. Its real indication, thus, seems more as a strengthening than repair material.

Another fully absorbable material is TGR™ (Matrix Surgical Mesh) composed of two synthetic fibre types (co-polymer glycolide-lactide trimethylene carbonate/lactide and trimethyl carbonate) with a multifilament structure [151]. Preclinical experience with this material seems adequate [152], although this has not been confirmed clinically [153].

Finally, another totally degradable material is Phasix™, a biosynthetic absorbable monofilament mesh (poly-4-hydroxybutyrate) [154,155,156,157]. This prosthesis has shown good outcomes in preclinical studies [158]. Its absorption over time is, however, disputed, as in some studies, material remains have been observed 18 months after implant [159]. Clinical trials are still scarce. The use of this material in the repair of ventral hernias has been associated with no recurrences after two years [160]. However, in another study examining its use for inguinal hernia repair, recurrence at 18 months post-implant was 9% [161].

## 5. Prosthetic Structure and Placement in Host Tissue: Adapting to Surgical Techniques

Regardless of its chemical composition, any prosthetic material of reticular structure (non absorbable, absorbable, or partially absorbable), needs to be implanted at a tissue/tissue interface. To avoid complications, these materials must not be placed in contact with a peritoneal interface. The selection of the reticular mesh to be used, i.e., high- or low density, will depend on patient factors such as obesity or physical requirements (physical demands). The latest generation fully absorbable reticular materials require longer-term follow up to assess their repair behaviour and efficacy. Surgical treatments with reticular prostheses may be conventional open procedures or the more recently introduced robotic surgery.

Laminar-structured prosthetic materials and composites can be placed at the peritoneal interface given their good behaviour in relation to the visceral peritoneum. An organized mesothelial deposit on these materials makes them ideal for placement at this interface. Surgical repairs with these materials can be laparoscopic and/or robotic.

## 6. Future Perspectives and Conclusions

The progressive use in recent years of biomaterials for hernia repair has led to their constant modification with the aim of obtaining a biomaterial showing optimal behaviour at every tissue interface. Despite such efforts, we still do not have the ideal prosthesis as it is proving difficult to generate a product able to adapt to all applications. Research and development has been evolving from simple tissue repair towards the actual regeneration of tissues, giving rise to new prosthetic materials that are fully biodegradable in the long term such that minimal foreign material is left behind in the host. Similarly, the development of functionalized materials as carriers of agents able to mitigate some complications, such as biomaterial infection, is today a priority line of investigation.

One of the main hurdles met when trying to elucidate the biological behaviour of prosthetic materials used for hernia repair is the difficulty in conducting investigations in humans. There are no markers related to the wound-repair process that could indicate which patients are at risk or not of showing poor repair. This demonstrates that experimental or preclinical studies are an important source of knowledge about some biological behaviours despite the biases these may entail.

## Figures and Tables

**Table 1:** Classification of the different types of polymeric meshes employed in hernia repair and the most representative modifications introduced. (PVDF, polyvinylidene fluoride; PLA, polylactic acid).

Type of Mesh	Modifications		Advantages	Limitations	References
Polyester (PO)	Standard mesh (Dacron^®^, Mersilene^®^)		Good and lasting mechanical strength	Adhesion formation Foreign body reaction	[13,14,15]
Polypropylene (PP)	Standard mesh (Marlex^®^, Prolene^®,^ Surgipro^®^)		Low recurrence rates Flexible and easily inserted Good mechanical resistance	High adhesion formation Disorganized neoperitoneum	[16,17,18,19,20,21,22,23,24,25,26,27,64,65,75]
	Structural modifications	Increased pore size Smaller knots Fine filaments Lower density (Parietene^TM^, Optilene^®^)	Improved integration and compliance Reduction of foreign material Reduction of inflammation and fibrosis Reduction of bridging effect	Adhesion formation	[50,51,52,53,54,55,56,69,70,72,73,74]
	Introduction of a second component	Absorbable filaments (Vypro^®,^ Ultrapro^®^)	Reduced foreign material	Adhesion formation	[57,71]
		Inert filaments: PVDF (Dynamesh^®^)	Diminished inflammatory response Resistance to degradation	Controversial results among experiments Adhesion formation	[93,94,95,96,97,98,99,100,101,102,103]
		Mesh coating: titanium (TiMESH^®^)	Improved biocompatibility Diminished foreign body reaction		[58,77,78,79,80,81,102]
	Self-gripping	PLA hooks (Progrip^®^) Adhesive (Lifemesh^TM^, Adhesix^®^)	Results comparable to sutured meses (Progrip^®^) Avoidance of the trauma caused by sutures or tacks	Mesh dislocated (onlay procedures)	[88,89,90,91,92]
Polytetrafluoroethylene (PTFE)	Expanded PTFE, laminar structure	Standard material (Soft Tissue Patch^®^)	Good biological tolerance Low incidence of adhesions Adequate neoperitoneum	Deficient tissue incorporation Reduced mechanical strength (vs. PP) Encapsulation Scarce angiogenesis	[30,31,32,33,34,35,36,37,38,39,40,41,42,43,59,64,65,66,67,68,75]
		Introduction of evenly spaced large pores (Mycro Mesh^®^)	More rapid tissue incorporation	Not mentioned	[44,60]
		Non-porous side and standard microporous surface (DualMesh^®^)	Adhesion prevention Good tissue ingrowth at microporous/rougher surface	Poor tissue integration at nonporous surface	[45,46,75]
		Rougher surface (Dual Mesh Corduroy^®^)			
		Pretreatment with antibacterial agent (Dual Mesh Plus^®^)	Reduced adherence of bacteria	Not mentioned	[46]
	PTFE, reticular structure (Infinit Mesh^®^)		Improved tissue incorporation Improved mechanical strength (vs. PTFE)	Adhesion formation High elastic modulus	[47,48,49,62]
	Condensed PTFE (MotifMESH^®^)		Reduced adhesion formation Minimal bacterial adherence	Adhesions on raised edges	[104,105,106,107]

**Table 2:** Classification of the different types of composite meshes employed in hernia repair and the most representative modifications introduced (PP, polypropylene; PO, polyester; PTFE, polytetrafluoroethylene; PU, polyurethane; PEG, polyethylene glycol; hy, hyaluronate; pd, polydioxanone; PGA, polyglycolic acid; P4H, poly-4-hydroxybutirate).

Type of Mesh	Modifications		Advantages	Limitations	References
Classic composite materials	Tissue integrating component	Reticular non absorbable mesh (PP, PO)	Good host tissue ingrowth Good mechanical strength Adequate behaviour at the peritoneal interface Reduced inflammatory reaction	Foreign material in the recipient	[108,109,110,111,112,113,114,115,116,117,118,119,120,121,122,123,124,125,126,127,128,129,130,131,132,133,134,135,136,137,138,139,140,141,142]
	Visceral component	Physical barrier (non absorbable): PTFE (Composix^®^) PU (PL-PU99^®^)			
		Chemical barrier (absorbable): PEG (Parietex Composite^TM^) hy (Sepramesh^TM^) pd + cellulose (Proceed^TM^)			
Last generation composites	Tissue integrating component	Partially or totally absorbable mesh: PP+PGA (Ventraligth ^TM^ ST) P4H (Phasix ^TM^ ST) Non absorbable mesh: PO (Symbotex^TM^) PP (Physiomesh^TM^)	Same as classic composites Reduced foreign material in the recipient	Not mentioned	[143,144,145,146,147]
	Visceral component	Chemical barrier (absorbable): pd hydrogel (Ventraligth^TM^) Collagen+chitosan (Symbotex^TM^) Polyglecaprone 25 (Physiomesh^TM^) PGA hydrogel (Phasix ^TM^ ST)			

## Data Availability

Not applicable.
